# Effect of Breast Milk Lead on Infant Blood Lead Levels at 1 Month of Age

**DOI:** 10.1289/ehp.6616

**Published:** 2004-05-11

**Authors:** Adrienne S. Ettinger, Martha María Téllez-Rojo, Chitra Amarasiriwardena, David Bellinger, Karen Peterson, Joel Schwartz, Howard Hu, Mauricio Hernández-Avila

**Affiliations:** ^1^Environmental Epidemiology Program, Department of Environmental Health, Harvard School of Public Health, Boston, Massachusetts, USA; ^2^Channing Laboratory, Department of Medicine, Brigham and Women’s Hospital, Harvard Medical School, Boston, Massachusetts, USA; ^3^Centro de Investigación de Salud Poblacional, Instituto Nacional de Salud Pública, Cuernavaca, Morelos, México; ^4^Department of Neurology, Children’s Hospital, Harvard Medical School, Boston, Massachusetts, USA; ^5^Departments of Nutrition,; ^6^Society, Human Development, and Health, and; ^7^Occupational Health Program, Department of Environmental Health, Harvard School of Public Health, Boston, Massachusetts, USA

**Keywords:** blood lead, breast milk lead, breast-feeding, KXRF bone lead, lactation

## Abstract

Nursing infants may be exposed to lead from breast milk, but relatively few data exist with which to evaluate and quantify this relationship. This route of exposure constitutes a potential infant hazard from mothers with current ongoing exposure to lead as well as from mothers who have been exposed previously due to the redistribution of cumulative maternal bone lead stores. We studied the relationship between maternal breast milk lead and infant blood lead levels among 255 mother–infant pairs exclusively or partially breast-feeding through 1 month of age in Mexico City. A rigorous, well-validated technique was used to collect, prepare, and analyze the samples of breast milk to minimize the potential for environmental contamination and maximize the percent recovery of lead. Umbilical cord and maternal blood lead were measured at delivery; 1 month after delivery (± 5 days) maternal blood, bone, and breast milk and infant blood lead levels were obtained. Levels of lead at 1 month postpartum were, for breast milk, 0.3–8.0 μg/L (mean ± SD, 1.5 ± 1.2); maternal blood lead, 2.9–29.9 μg/dL (mean ± SD, 9.4 ± 4.5); and infant blood lead, 1.0–23.1 μg/dL (mean ± SD, 5.5 ± 3.0). Infant blood lead at 1 month postpartum was significantly correlated with umbilical cord (Spearman correlation coefficient *r_S_* = 0.40, *p* < 0.0001) and maternal (*r_S_* = 0.42, *p* < 0.0001) blood lead at delivery and with maternal blood (*r_S_* = 0.67, *p* < 0.0001), patella (*r_S_* = 0.19, *p* = 0.004), and breast milk (*r_S_* = 0.32, *p* < 0.0001) lead at 1 month postpartum. Adjusting for cord blood lead, infant weight change, and reported breast-feeding status, a difference of approximately 2 μg/L (ppb; from the midpoint of the lowest quartile to the midpoint of the highest quartile) breast milk lead was associated with a 0.82 μg/dL increase in blood lead for breast-feeding infants at 1 month of age. Breast milk lead accounted for 12% of the variance of infant blood lead levels, whereas maternal blood lead accounted for 30%. Although these levels of lead in breast milk were low, they clearly have a strong influence on infant blood lead levels over and above the influence of maternal blood lead. Additional information on the lead content of dietary alternatives and interactions with other nutritional factors should be considered. However, because human milk is the best and most complete nutritional source for young infants, breast-feeding should be encouraged because the absolute values of the effects are small within this range of lead concentrations.

Breast milk has been suggested as a significant potential source of lead exposure to nursing infants (Silbergeld 1991), but relatively few data exist with which to evaluate and quantify this relationship. This phenomenon constitutes a potential public health problem in countries where environmental lead exposure is continuing as well as in countries where environmental lead exposure has declined (Abadin et al. 1997). Previously, we reported that maternal blood and bone lead levels are both important determinants of lead in breast milk (Ettinger et al. 2004). Lead from current maternal exposure, as well as that accumulated in bone from past environmental exposures and subsequently released into blood, is excreted into breast milk and thus may be ingested by the nursing infant.

Studies of lead in human milk have found concentrations ranging over three levels of magnitude from < 1 to > 100 μg/L (ppb) (Gulson et al. 1998; Namihira et al. 1993). However, there are limited epidemiologic data available regarding the potential exposure that this represents for the breast-feeding infant.

There are some data from rodents on the lactational transfer and uptake of lead in the newborn. Kostial and Momcilovic (1974) showed that the peak transfer of radiolabeled lead in mice from mother to litter occurred during lactation. Keller and Doherty (1980) found that 25% of maternal bone lead burden in mice was transferred to infant mice, and most of this activity occurred during lactation. Mouse breast milk was found to concentrate lead at around 25 times the level circulating in plasma. Amount of lead transferred seems to vary considerably by species (Oskarsson et al. 1995); however, there may be more efficient absorption of lead by the neonate compared with the adult (Oskarsson et al. 1998; Palminger Hallén et al. 1996).

In humans, Rabinowitz et al. (1985) described a log-linear dose–response relationship between breast milk lead and infant blood lead at 6 months of age (β = 3.0 μg/dL, SE = 1.1 μg/dL, *r*^2^ = 10%, *p* = 0.009). By examining the lead isotopic ratios in a small number of infants born to recent immigrants to Australia (and infants of Australian controls), Gulson et al. (1998) found that for the first 60–90 days postpartum the contribution from breast milk to blood lead in the infants varied from 36 to 80%.

We evaluated the effect of breast milk lead on infant blood lead levels to quantify the dose–response relationship in a large, population-based sample of infants exclusively or partially breast-fed through 1 month of age. We used a rigorous, well-validated technique to collect, prepare, and analyze the samples of breast milk to minimize the potential for contamination and maximize the percent recovery of lead.

## Materials and Methods

We conducted a cross-sectional study of 255 nursing infants at 1 month postpartum in Mexico City. Subjects included infants born to a subcohort of women recruited for later participation in a randomized placebo-controlled trial of calcium supplementation during lactation. Informed consent, questionnaire information, and samples for the present study were obtained before the initiation of calcium supplementation. All participating mothers received a detailed explanation of the study and counseling on reduction of lead exposure. The research protocol was approved by the human subjects committees of the National Institute of Public Health of Mexico, Harvard School of Public Health, and the participating hospitals.

Data collection methods have been described in detail elsewhere (Hernández-Avila et al. 2003). Between January 1994 and June 1995, 2,945 potential study participants were interviewed at three maternity hospitals in Mexico City. Of these, 1,398 were eligible for the trial. From the women identified as eligible, 629 (45%) agreed to participate in the study. These women completed a baseline evaluation including a questionnaire that assessed known risk factors for environmental lead exposure, dietary assessment of nutrient intake, and breast-feeding practices. At 1 month post-partum (± 5 days), field personnel visited study participants at home to obtain anthropometric measurements, blood, and breast milk samples. Maternal bone lead was estimated by K X-ray fluorescence (KXRF) at the research facility at the American British Cowdray (ABC) Hospital. Three hundred ten samples of breast milk from the 1 month postpartum visit were analyzed for lead content. This report is limited to the 255 subjects with both breast milk and infant blood lead levels available at 1 month postpartum.

### Blood lead.

Blood lead measurements were performed using graphite furnace atomic absorption spectrophotometry (model 3000; PerkinElmer, Norwalk, CT, USA) at the ABC Hospital Trace Metal Laboratory according to a technique described by Miller et al. (1987). The laboratory participates in the Centers for Disease Control and Prevention blood lead proficiency testing program administered by the Wisconsin State Laboratory of Hygiene (Madison, WI, USA). The laboratory standardization program provided external quality control specimens varying from 2 to 88 μg/dL. Our laboratory maintained acceptable precision and accuracy over the study period (correlation = 0.98; mean difference = 0.71 μg/dL; SD = 0.68).

### Bone lead.

We used a spot-source ^109^Cd KXRF instrument constructed at Harvard University and installed at the research facility in Mexico City to measure maternal bone lead. Thirty-minute *in vivo* measurements of each subject’s mid-tibial shaft (representing cortical bone) and patella (trabecular bone) were obtained after each region had been washed with a 50% solution of isopropyl alcohol. The physical principles, technical specifications, validation, and use of the KXRF technique have been described in detail elsewhere (Hu et al. 1991). The instrument provides an estimate of the uncertainty associated with each measurement. For quality control, we excluded bone lead measurements with uncertainty estimates that were > 10 and 15 μg lead/g mineral bone for tibia (*n* = 12) and patella (*n* = 38), respectively, from the entire cohort of 629 women. These measurements generally reflect excessive patient movement outside the measurement field or excessive thickness of overlaying tissue and do not produce acceptable results.

### Breast milk lead.

Breast milk samples were collected at 1 month postpartum from lactating women using techniques to minimize potential for environmental contamination. Before manual expression of milk, the breast was washed with deionized water, which also was collected and analyzed for lead contamination. Ten milliliters of milk was collected in preleached polypropylene tubes. Samples were frozen, shipped to the Channing Laboratory, and stored at −30°C (Fisher IsoTempPlus, New York, NY, USA) until analysis.

Breast milk sample preparation was performed at University Research Institute for Analytical Chemistry (Amherst, MA, USA), and instrumental analysis was performed at the Trace Metals Laboratory of Harvard School of Public Health. Digestion was performed using HNO_3_ acid in high-temperature high-pressure asher (HPA; Anton Paar USA, Ashland, VA, USA). Lead content in the samples was analyzed by isotope dilution–inductively coupled plasma mass spectrometry (ID-ICPMS; Sciex Elan 5000; PerkinElmer,) by methods previously described in detail (Ettinger et al. 2004). The limit of detection for lead analysis in breast milk by HPA digestion and ID-ICPMS is 0.1 ng/mL (ppb) milk.

### Statistical analysis.

Univariate and bivariate summary statistics and distributional plots were examined for all variables. Infant blood lead levels were highly positively skewed, so for the subsequent regression analyses, the log (base *e*)-transformed values of the dependent variable were used. Possible associations between infant blood lead and the independent variables were separately explored with bivariate linear regression models. Spearman correlation coefficients with *p*-values are reported. Characteristics of the participants were compared by reported breast-feeding practice (partial vs. exclusive) using Wilcoxon sign rank/chi-square tests of equality of two sample population means/proportions. Extreme values of infant blood lead (*n* = 3) and breast milk lead (*n* = 9) were identified using the generalized extreme studentized deviation many-outlier procedure (Rosner 1983) and excluded from the multivariate regression analyses. We used multiple linear regression models to describe the relationships between infant blood lead, breast milk lead, and the covariates of interest, which were determined *a priori* based on biologic considerations. Infant weight change (weight at 1 month minus birth weight) was used as a surrogate for the amount of breast milk consumed. The final model for infant blood lead included breast milk lead, umbilical cord lead at delivery, breast-feeding status (exclusive vs. partial), and infant weight change. Breast milk lead was divided into quartiles, and the midpoint of the quartile was used to predict the infant blood lead level for exposure at that level based on the final model for infant blood lead. To explore potential nonlinear associations between breast milk lead and infant blood lead levels, we examined the relations between the variables using generalized additive models. All statistical analyses were performed using Statistical Analysis System (SAS) software (release 8.01; SAS Institute, Inc., Cary, NC, USA) and S-PLUS (6.0 professional edition for Windows; Insightful Corp., Seattle, WA, USA).

## Results

**Figure 1:**
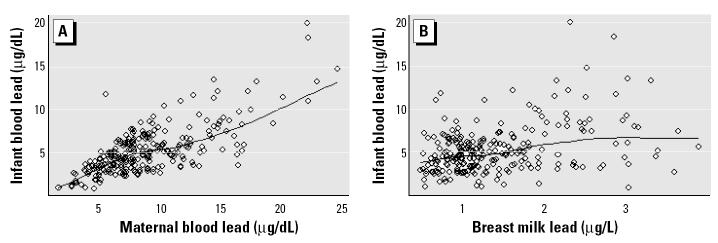
Smooth scatter plots (Lowess; bandwidth = 0.75) of infant blood lead by (*A*) maternal blood lead and (*B*) breast milk lead at 1 month postpartum.

Summary statistics for the lead biomarkers of mothers and infants in the study (*n* = 255) are shown in Table 1. Levels of lead in breast milk ranged from 0.3 to 8.0 μg/L (ppb). Infant blood lead levels (mean ± SD) were 5.5 ± 3.0 μg/dL and ranged from 1.0 to 23.1 μg/dL. Figure 1 shows the unadjusted relationships of maternal blood lead and breast milk lead on infant blood lead levels at 1 month postpartum. Infant blood lead at 1 month postpartum was significantly correlated with umbilical cord (Spearman correlation coefficient *r_S_* = 0.40, *p* < 0.0001) and maternal (*r_S_* = 0.42, *p* < 0.0001) blood lead at delivery and with concurrent maternal blood (*r_S_* = 0.67, *p* < 0.0001), patella (*r_S_* = 0.19, *p* = 0.004), and breast milk (*r_S_* = 0.32, *p* < 0.0001) lead at 1 month postpartum (Table 2).

On average, mothers in the study were 24.3 years of age (range, 14–40 years of age) and had lived in Mexico City for 20 years (range, 0.5–40 years). Forty percent of women were primiparous. Of the 152 women with prior pregnancies, 22% (*n* = 55) had completed 12 or more months of total breast-feeding of their previous infants.

Differences in maternal and infant characteristics by reported breast-feeding practice (exclusive *n* = 88 vs. partial *n* = 165) at 1 month postpartum are shown in Table 3. Breast milk lead levels (mean μg/L ± SD) were similar (*p* = 0.84) among women who reported practicing exclusive breast-feeding (1.4 ± 1.1) compared with women who practiced partial lactation (1.5 ± 1.2). With respect to other subject characteristics, subjects differed somewhat by lead-glazed ceramics use. Subjects who were exclusively breast-feeding at 1 month postpartum were less likely to have used lead-glazed ceramics to store, prepare, or serve food in the past (*p* = 0.03), with 69% of women reporting past use of lead-glazed ceramics compared with 81% of partially breast-feeding mothers. In addition, those subjects who were partially breast-feeding reported slightly higher, although not statistically significant, current use of lead-glazed ceramics (*p* = 0.08). However, exclusively breast-feeding women (10%) were more likely to have reported current smoking or smoking during pregnancy than were partially breast-feeding women (3.6%; *p* = 0.03). Partially breast-feeding women were more likely to be married (74 vs. 61%, *p* = 0.04) and reported slightly higher dietary calcium intake (1,193 vs. 1,036 mg, *p* = 0.002) than were women who were exclusively breast-feeding at 1 month postpartum.

**Figure 2:**
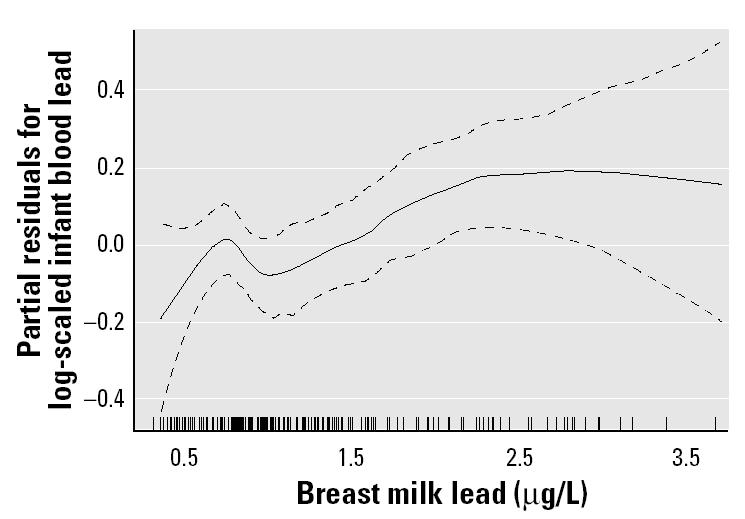
Generalized additive model-adjusted dose–response function for log-scaled infant blood lead and breast milk lead concentrations at 1 month postpartum adjusted for umbilical cord blood lead, infant weight change, and breast-feeding practice. The dashed lines represent 95% pointwise confidence intervals.

Figure 2 shows the nonparametric dose–response relationship of maternal blood lead and breast milk lead on infant blood lead levels at 1 month postpartum from the generalized additive model, adjusted for umbilical cord blood lead (micrograms per deciliter), infant weight change (grams), and breast-feeding practice (exclusive vs. partial).

**Figure 3:**
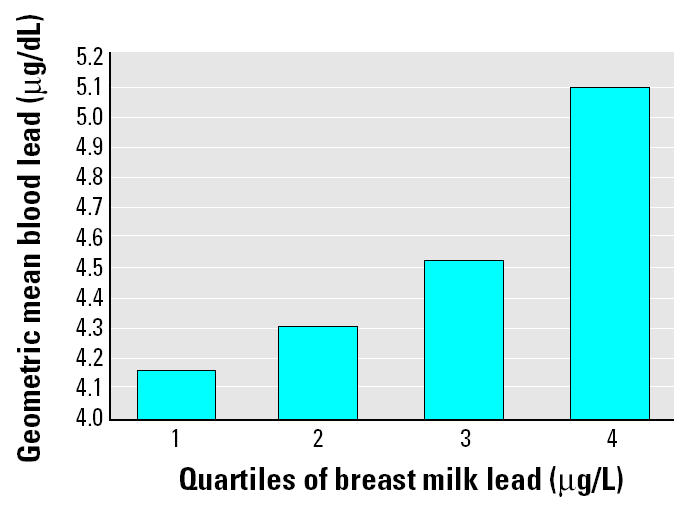
Geometric mean infant blood lead level (μg/dL) predicted at each level of breast milk lead. The midpoints of quartile 1 = 0.53 μg/L; quartile 2 = 0.83; quartile 3 = 1.28; quartile 4 = 2.34. A difference of approximately 2 μg/L (ppb; from the midpoint of the lowest quartile to the midpoint of the highest quartile) breast milk lead was associated with a 0.82-μg/dL increase in blood lead for breast-feeding infants at 1 month of age.

In multivariate linear regression models, breast milk was a significant predictor (*p* = 0.02) of infant blood lead after controlling for umbilical cord lead, infant weight change, and breast-feeding practice. Breast milk accounted for 12% of the variance of infant blood lead levels (Table 4), whereas maternal blood lead accounted for 30% of the variance of infant blood lead levels in a similar model (data not shown). To predict the effect of breast milk lead on infant blood lead level, we calculated infant blood lead for each quartile of breast milk exposure based on the final model. Adjusting for cord blood lead, infant weight change, and reported breast-feeding practice, we found that a difference of approximately 2 μg/L (from the midpoint of the lowest quartile to the midpoint of the highest quartile) of breast milk lead was associated with a 0.82-μg/dL increase in blood lead for infants at 1 month of age (Figure 3). This effect was almost identical among the exclusive and partial breast-feeding groups, so the combined data are presented.

## Discussion

From birth to 6 months, the infant’s exposure to lead is typically dominated by dietary sources. Although the levels of lead in breast milk reported here were low, they clearly had a strong influence on infant blood lead levels over and above the influence of maternal blood lead. In our study, breast milk lead accounted for 12% of the variance of infant blood lead levels at 1 month of age. In the only other large-scale study of breast milk and infant blood lead levels, milk lead accounted for 10% of the variance in 6-month blood lead (Rabinowitz et al. 1985).

It is important to estimate the contribution from the non–breast milk sources to total lead exposure from dietary intake. Rabinowitz et al. (1985) found breast milk to be the strongest correlate of 6-month blood leads, whereas formula lead correlated poorly with infant blood lead levels. Gulson et al. (1998) showed that the contribution of formula to infant blood lead varied from 24 to 68% in formula-fed infants. Therefore, it would have been important to document the sources and amount of lead in diet (other than from breast milk) consumed by infants in this population.

Our study was completed during the voluntary removal of lead soldered cans from the market in Mexico (De León 1996), so lead in canned infant formula may have been an additional source. We can only speculate that the contribution to lead exposure from foods and beverages used as alternatives to or in combination with breast milk may have been similar to or greater than that of breast milk. Although there may be more lead in infant formula, the relative bioavailability of such lead may be less than that of lead in breast milk. For example, it has been documented that iron is more readily absorbed from breast milk than from infant formula (Lonnerdal 1985).

Estimating the potential lead dose to infants from breast milk requires information about the quantity of breast milk consumed per day and the duration over which breast-feeding occurs (U.S. EPA 1997). Average intakes are about 750–800 g/day (range, 450–1,200 g/day) for the first 4–5 months of life [Institute of Medicine (IOM) 1991]. However, infant birth weight and nursing frequency have been shown to influence the rate of intake (IOM 1991). We attempted to control for consumption using infant weight change from birth to 1 month as a surrogate in our analyses.

It may also be important to estimate the contribution from the nondietary sources of lead to total body burden of young children. Although it is widely assumed that infant exposures to lead during the first 4–6 months of life are derived from diet, Manton et al. (2000) showed that lead dust contributed to exposure in U.S. infants in the first 4 months of life. However, lead dust is not a common source of exposure in Mexico. Also, neonatal bone turnover is a potential endogenous source of lead in infant blood (Gulson et al. 2001).

Our previous research (Hernández-Avila et al. 1996; Hu et al. 1996) and the research of others (Gulson et al. 1997; Rothenberg et al. 2000) have clearly shown that maternal bone stores of lead are mobilized to a marked degree during lactation. Breast-feeding practices and maternal bone lead are important predictors of maternal blood lead levels over the course of lactation (Téllez-Rojo et al. 2002). Previously, we reported that maternal blood and bone lead levels are both important determinants of lead in breast milk (Ettinger et al. 2004). Our data suggest that despite the potential for lead exposure, even among this population of women who have been relatively highly exposed, levels of lead in breast milk are low. However, we have demonstrated here that breast milk lead levels are highly influential on infant blood lead levels at 1 month of age. This is a cross-sectional analysis at 1 month postpartum and cannot evaluate changes in breast milk, infant blood, and bone lead levels over the course of lactation. It will be important to determine whether the degree of this influence changes over the course of lactation.

Due to the unique nutritional characteristics of human milk, breast-feeding is thought to be the optimal mode of nutrient delivery to term infants [American Academy of Pediatrics 1997; IOM 1991; World Health Organization (WHO) 1995]. Better understanding of the potential for neonatal exposure, including kinetics in the lactating mother and knowledge about alternative dietary sources of lead, is needed for risk assessment. Given the correlation of breast milk lead levels with maternal and infant blood lead levels, milk lead can be used as an indicator of both maternal and neonatal exposure (Hallén et al. 1995). Additional information on the lead content of dietary alternatives should be investigated in comparison with breast milk levels in a specific population and interactions with other nutritional factors should also be considered. This highlights the need to further investigate interventions that may reduce lead exposure from endogenous sources. Because bone lead has a half-life of years to decades, infants will continue to be at risk for exposure long after environmental sources of lead have been abated. In addition, efforts to reduce ongoing environmental exposure to lead should be continued, and ways to mitigate the effects of past exposures should be investigated. However, because human milk is the best and most complete nutritional source for young infants, breast-feeding should be encouraged because the absolute values of the effects are small within this range of lead concentrations.

## Figures and Tables

**Table 1:** Summary statistics for lead biomarkers among mothers and infants in the study.

Biomarker of lead exposure	No.	Mean ± SD	Minimum	Maximum
At delivery				
Maternal blood lead (μg/dL)	251	8.7 ± 4.2	2.1	23.7
Umbilical cord lead (μg/dL)	222	6.7 ± 3.6	1.2	26.3
At 1 month postpartum				
Breast milk lead (μg/L)	255	1.5 ± 1.2	0.3	8.0
Maternal blood lead (μg/dL)	255	9.4 ± 4.5	1.8	29.9
Maternal patella lead (μg/g)*^a^*	246	15.3 ± 15.0	< 1	67.2
Maternal tibia lead (μg/g)*^a^*	250	10.0 ± 10.4	< 1	76.6
Infant blood lead (μg/dL)	255	5.5 ± 3.0	1	23.1

a Includes measurements with negative values: patella (*n* = 37), tibia (*n* = 34).

**Table 2:** Correlation matrix for lead biomarkers.*^a^*

	At delivery		At 1 month postpartum				
Biomarker of lead exposure	Umbilical cord (*n* = 222)	Maternal blood (*n* = 220)	Breast milk (*n* = 255)	Maternal blood (*n* = 255)	Maternal patella (*n* = 246)	Maternal tibia (*n* = 250)	Infant blood (*n* = 255)
At delivery							
Umbilical cord	1.00	0.82	0.34	0.51	0.019	0.12	0.40
		*p* < 0.0001	*p* < 0.0001	*p* < 0.0001	*p* = 0.006	*p* = 0.07	*p* < 0.0001
Maternal blood		1.00	0.36	0.56	0.22	0.18	0.42
			*p* < 0.0001	*p* < 0.0001	*p* = 0.0006	*p* = 0.006	*p* < 0.0001
At 1 month postpartum							
Breast milk			1.00	0.42	0.14	−0.005	0.32
				*p* < 0.0001	*p* = 0.03	*p* = 0.94	*p* < 0.0001
Maternal blood				1.00	0.30	0.19	0.67
					*p* < 0.0001	*p* < 0.0001	*p* < 0.0001
Maternal patella					1.00	0.27	0.19
						*p* < 0.0001	*p* = 0.004
Maternal tibia						1.00	0.08
							*p* = 0.2
Infant blood							1.00

a Spearman correlation coefficients; prob > |*r*| under H_0_; rho = 0.

**Table 3:** Maternal and infant characteristics by reported breast-feeding practice.

	Reported breast-feeding practice				
	Exclusive lactation		Partial lactation		
Characteristic	No.	Mean ± SD	No.	Mean ± SD	*p*-Value*^a^*
At delivery					
Umbilical cord lead (μg/dL)	78	6.4 ± 2.9	143	6.9 ± 3.9	0.26
Maternal blood lead (μg/dL)	86	8.1 ± 3.8	164	9.0 ± 4.4	0.09
Infant birth weight (g)	88	3,140 ± 372	165	3,121 ± 380	0.71
Infant birth length (cm)	86	50.6 ± 2.1	162	50.3 ± 2.3	0.30
Infant head circumference (cm)	84	34.0 ± 1.4	157	33.9 ± 1.4	0.45
At 1 month of age (infant)					
Blood lead (μg/dL)	88	5.4 ± 3.2	165	5.6 ± 3.0	0.54
Weight (g)	87	4,263 ± 516	165	4,178 ± 534	0.22
Length (cm)	88	53.5 ± 2.1	165	53.6 ± 2.0	0.79
At 1 month postpartum (maternal)					
Breast milk lead (μg/L)	88	1.4 ± 1.1	165	1.5 ± 1.2	0.85
Blood lead (μg/dL)	88	9.4 ± 4.8	165	9.5 ± 4.3	0.82
Patella lead (μg/g)	85	15.4 ± 12.6	159	15.4 ± 16.1	0.98
Tibia lead (μg/g)	87	9.9 ± 9.5	164	10.0 ± 10.9	0.96
Age (years)	88	24.6 ± 5.4	165	24.2 ± 4.7	0.54
Years living in Mexico City	88	19.2 ± 9.4	165	20.7 ± 8.2	0.20
Education (years)	85	8.8 ± 3.1	165	9.2 ± 3.0	0.30
Married (%)	88	61	165	74	0.04
Estimated calcium intake (mg)	88	1,036 ± 358	164	1,193 ± 397	0.002
Previous lactation > 12 months (%)	88	28.4	165	18.2	0.06
No. of pregnancies	88	2.2 ± 1.3	165	2.0 ± 1.2	0.22
Primiparous (%)	88	35.2	165	43.6	0.19
Current use of lead-glazed ceramics (%)	88	34.1	165	45.5	0.08
Past use of lead-glazed ceramics (%)	88	69.3	165	81.2	0.03
Current smoking or during pregnancy (%)	88	10.2	165	3.6	0.03

a *p*-Value from Wilcoxon sign rank test/chi-square test of equality of two sample population means/proportions.

**Table 4:** Multivariate regressions for infant blood lead.*^a^*

	β-coefficient	SE	*p*-Value	Partial *R*^2*^b^*^
Intercept	1.06	0.15	< 0.0001	—
Breast milk lead*^c^* (μg/L)	0.10	0.04	0.02	0.12
Umbilical cord blood lead (μg/dL)	0.05	0.009	< 0.0001	0.11
Infant weight change (g)	−0.00009	0.00007	0.2	0.007
Breast-feeding practice*^d^*	0.09	0.06	0.15	0.015

a Infant blood lead levels log (base *e*) transformed, *n* = 3 extreme outliers excluded.

b Adjusted model *R*^2^ = 0.2259.

c Breast milk lead *n* = 9, extreme outliers removed.

d Exclusive lactation = reference group.
